# Flavonoids as Modulators of Neuroinflammation in Affective Disorders: A Narrative Review

**DOI:** 10.3390/ijms27104561

**Published:** 2026-05-19

**Authors:** Gilberto Uriel Rosas-Sánchez, Rosa Rodríguez-Yoval, León Jesús German-Ponciano, Oscar Gutiérrez-Coronado, Paola Trinidad Villalobos Gutiérrez, Rafael Fernández-Demeneghi, Alma Gabriela Martínez-Moreno, José Luis Muñoz-Carrillo, Cesar Soria-Fregozo

**Affiliations:** 1 Programa de Estancias Posdoctorales por México, Secretaría de Ciencia, Humanidades, Tecnología e Innovación SECIHTI, Centro Universitario de Los Lagos, Universidad de Guadalajara, Lagos de Moreno 47460, Jalisco, Mexico; yovalro@hotmail.com (R.R.-Y.); mcbjlmc@gmail.com (J.L.M.-C.); 2 Departamento de Ciencias de la Tierra y de la Vida, Centro Universitario de Los Lagos, Universidad de Guadalajara, Lagos de Moreno 47460, Jalisco, Mexico; 3 Laboratorio de Neurofarmacología, Instituto de Neuroetología, Universidad Veracruzana, Xalapa 91190, Veracruz, Mexico; lgerman@uv.mx; 4 Laboratorio de Inmunología, Centro Universitario de los Lagos, Universidad de Guadalajara, Lagos de Moreno 47460, Jalisco, Mexico; oscar.gcoronado@academicos.udg.mx (O.G.-C.); paola.villalobos2452@academicos.udg.mx (P.T.V.G.); 5 Instituto de Investigaciones en Comportamiento Alimentario y Nutrición, Universidad de Guadalajara, Ciudad Guzmán 49000, Jalisco, Mexico; rafael_demeneghi@hotmail.com (R.F.-D.); alma.martinez@cusur.udg.mx (A.G.M.-M.)

**Keywords:** flavonoids, neuroinflammation, animal model, bipolar disorder, anxiety, depression, neuroprotection

## Abstract

Affective disorders, including anxiety, depression, and bipolar disorder (BD), represent a global mental health burden with complex, multifactorial etiopathogenesis. Increasing evidence implicates neuroinflammation, oxidative stress, and dysregulation of neurotrophic and neurotransmitter systems as central mechanisms driving these conditions. Flavonoids, a structurally diverse class of plant-derived polyphenolic compounds abundantly found in fruits, vegetables, tea, and other dietary sources, have emerged as promising modulators of these pathophysiological pathways. This narrative review synthesizes current preclinical and clinical evidence on the role of flavonoids and related natural compounds in modulating neuroinflammation and affective disorders. We describe the major flavonoid subclasses—flavones, flavonols, isoflavones, anthocyanins, flavanones, and flavan-3-ols—and analyze their mechanisms of action, including inhibition of the NF-κB/NLRP3 axis, reduction in pro-inflammatory cytokines, attenuation of oxidative stress via Nrf2 pathway activation, modulation of monoaminergic and GABAergic neurotransmission, promotion of Brain-Derived Neurotrophic Factor (BDNF)-mediated neuroplasticity, and regulation of the microbiota–gut–brain axis. Preclinical studies consistently demonstrate anxiolytic and antidepressant effects for compounds such as quercetin, luteolin, apigenin, and chrysin; however, clinical evidence remains limited and methodologically heterogeneous. Future research should prioritize bioavailability-enhanced formulations, standardized clinical trials, and biomarker-guided stratification to fully establish the therapeutic potential of flavonoids in affective disorders.

## 1. Introduction

Given the fundamental role of neuroinflammation in affective disorders, there is growing interest in natural compounds, particularly flavonoids and polyphenols, for their potential to modulate inflammation and support brain function [1]. These compounds, primarily derived from plants, offer a multi-target approach to neuroprotection and mood regulation.

Flavonoids are a diverse class of plant-derived polyphenols found abundantly in fruits, vegetables, wine, tea, and cocoa [2,3,4]. Their beneficial effects on brain health are increasingly recognized due to their ability to cross the brain–blood barrier (BBB). Many flavonoids can penetrate the BBB and reach brain tissue, where they exert their effects [5,6,7]. This is a crucial characteristic for any compound intended to act on disorders of the central nervous system (CNS). These compounds possess potent antioxidant properties, allowing them to protect neurons against neurotoxin-induced damage and reduce oxidative stress, which is frequently implicated in neuroinflammation and neurodegenerative processes [1,8].

Flavonoids are potent modulators of neuroinflammation. Their mechanisms include the inhibition of pro-inflammatory mediators, as they can suppress the production and release of pro-inflammatory cytokines such as tumor necrosis factor alpha (TNF-α), interleukin-1β (IL-1β), and interleukin-6 (IL-6) by activated microglia and astrocytes [3,9,10]. Furthermore, they can modulate key inflammatory pathways, such as the NF-κB, MAPK, and JAK/STAT pathways, suppress the activation of the NLRP3 inflammasome, and influence glial cell polarization, promoting an anti-inflammatory state [3]. Some flavonoids can also increase the secretion of anti-inflammatory factors [3].

Several studies have shown that polyphenols, including flavonoids, promote brain plasticity and improve cognition [6]. They induce the production of BDNF, which is essential for neuronal growth, survival, and plasticity [1,9]. Additionally, they act as reversible inhibitors of monoamine oxidase (MAOIs), thereby modulating mood by increasing levels of dopamine (DA), serotonin (5-HT), and norepinephrine (NE) in the brain [6].

Specific flavonoids such as quercetin, luteolin, apigenin, phloretin, epigallocatechin-3-O-gallate, and cyanidin-3-O-glucoside have demonstrated neuroprotective and anti-inflammatory effects in preclinical and clinical models [3,11]. These compounds have shown promise in alleviating neuroinflammation in various brain disorders, including anxiety, BD and depression [3]. In addition to flavonoids, other natural compounds and phytochemicals also exhibit beneficial effects on mental health and brain function by targeting neuroinflammation. For example, curcumin, found in turmeric, has demonstrated neuroprotective, anti-inflammatory, antioxidant, and antidepressant effects [1,12]. Ginsenosides from ginseng promote neuroprotection, in part through mechanisms mediated by the gut microbiota [12]. *Ginkgo biloba* polysaccharides and lavender essential oil have shown improvements in neurotransmitter regulation, intestinal barrier integrity, and depression-like behaviors in preclinical models [12]. The ability of these natural compounds to act simultaneously on multiple pathophysiological pathways neuroinflammation, oxidative stress, neurotransmitter dysregulation, and neuroplasticity makes them attractive candidates for therapeutic intervention in affective disorders [1]. They are generally considered safe and non-invasive, offering a potential complementary treatment strategy, especially for conditions such as depression, where current therapies have limitations or side effects [7].

The objective of this narrative review is to synthesize and critically evaluate the existing scientific literature on the complex relationship between affective disorders (anxiety, depression, and BD), neuroinflammation, and the therapeutic potential of flavonoids and other natural compounds. It aims to elucidate the mechanisms by which neuroinflammation contributes to the pathophysiology of these affective disorders and to highlight the anti-inflammatory, antioxidant, and neuroprotective properties of flavonoids and other natural compounds as a basis for future therapeutic development.

## 2. Method

### 2.1. Design

This study was designed as a narrative literature review on flavonoids and natural compounds as modulators of neuroinflammation in affective disorders such as anxiety and depression. It analyzed information from scientific papers, book chapters, books, and official websites to provide an informative, critical, and useful synthesis of the topic. In a narrative review, there are no predetermined research questions or specific search strategies, only a topic of interest. Recognizing that narrative reviews lack systematic methods for identifying, evaluating, and synthesizing information which could lead authors to include or exclude information to support a particular position we describe the parameters used to include or exclude studies to ensure objective inclusion of information. These parameters included conducting a search and identifying keywords, reviewing abstracts and articles, and documenting results.

### 2.2. Criteria

The inclusion criteria focused on research articles, reviews, and statements that investigated the effects of flavonoids in preclinical and clinical studies on neuroinflammation and affective disorders, as well as their mechanisms of action, in English-language articles. Exclusion criteria included studies without full-text access, websites not affiliated with recognized academic, governmental, or institutional organizations (such as personal blogs, non-peer-reviewed online platforms, and commercial or promotional websites without scientific endorsement), duplicate publications, and doctoral dissertations.

### 2.3. Article Research

Data on the topic described in the inclusion criteria were searched in specialized databases such as PubMed, ScienceDirect, Web of Science, and Scopus using a combination of specific terms including “anxiety”, “depression”, “neuroinflammation”, “flavonoids”, “natural products”, “anxiolytic”, “antidepressant”, “treatment”, “animal”, “models”, “clinical studies”, and “flavonoids mechanism of action”.

## 3. Bipolar Disorder and Flavonoids: Neuroinflammatory Mechanisms and Therapeutic Evidence

BD is a chronic, disabling psychiatric condition characterized by recurrent episodes of mania, hypomania, and depression. It affects approximately 1–4% of the global population and is associated with significant functional impairment and increased mortality [13,14]. Although its etiology is multifactorial, converging evidence from clinical, neuroimaging, and post-mortem studies has established neuroinflammation and oxidative stress as central pathophysiological mechanisms in BD [15,16]. This understanding has opened new avenues for exploring natural anti-inflammatory compounds, particularly flavonoids, as potential modulators of the neurobiological alterations underlying this disorder.

### 3.1. Neuroinflammation and Oxidative Stress in Bipolar Disorder

Patients with BD consistently show elevated serum and cerebrospinal fluid levels of pro-inflammatory cytokines, including IL-6, TNF-α, IL-1β, and IL-18, during both manic and depressive phases, with the highest inflammatory burden observed during acute manic episodes [17,18]. Meta-analyses have confirmed increased IL-6, TNF-α, and soluble IL-2 receptor (sIL-2R) in BD, regardless of mood state, suggesting a persistent inflammatory endophenotype [18,19]. Microglial activation has been demonstrated in post-mortem brain tissue of BD patients, particularly in the prefrontal cortex, anterior cingulate cortex, and hippocampus—regions critically involved in mood regulation and executive function [20,21]. Reactive astrogliosis and reduced astrocyte density have also been documented, contributing to impaired glutamate reuptake, excitotoxicity, and disrupted synaptic homeostasis [15].

Oxidative stress is another hallmark of BD. Patients display increased levels of lipid peroxidation markers (e.g., MDA, 4-hydroxynonenal), elevated reactive oxygen and nitrogen species, and reduced activities of antioxidant enzymes including SOD, CAT, and GPx, primarily measured in peripheral blood (serum and plasma) and, in some studies, in postmortem brain tissue including the prefrontal cortex, hippocampus, and anterior cingulate cortex [22]. A meta-analysis of 44 studies (n = 3767) confirmed that BD is associated with significantly higher TBARS, MDA, and total nitrites in peripheral blood samples, along with lower glutathione levels in both peripheral blood and brain tissue compared to healthy controls [22]. Mitochondrial dysfunction, evidenced by impaired electron transport chain activity and decreased mitochondrial membrane potential, further amplifies oxidative damage and intersects with neuroinflammatory cascades through the NLRP3 inflammasome and NF-κB pathways [23,24]. Collectively, these findings support a model in which the interplay between neuroinflammation, oxidative stress, and mitochondrial dysfunction drives neuroprogression in BD, resulting in cumulative cognitive decline and increased episode frequency [25].

### 3.2. Preclinical Evidence of Flavonoids in Models Relevant to Bipolar Disorder

Although specific animal models of BD remain methodologically challenging, several pharmacological and genetic paradigms—including amphetamine-induced mania, ouabain-induced mania, and lithium–pilocarpine models—have been used to evaluate the neuroprotective effects of flavonoids in conditions that mimic manic and depressive states [26].

Quercetin has shown significant promise in preclinical BD-relevant models. In amphetamine- and methylphenidate-induced hyperactivity models in mice, quercetin administration (10–40 mg/kg) significantly attenuated locomotor hyperactivity, reduced oxidative stress markers in the prefrontal cortex and hippocampus, and decreased lipid peroxidation [27,28]. Its mechanisms include inhibition of NF-κB activation, suppression of the NLRP3 inflammasome, and upregulation of Nrf2-mediated antioxidant defenses—pathways consistently dysregulated in BD [29]. Additionally, quercetin was found to relieve D-amphetamine-induced manic-like behavior through activation of TREK-1 potassium channels in the prefrontal cortex [30]. Resveratrol, a non-flavonoid polyphenol with overlapping mechanisms, has also demonstrated mood-stabilizing effects in amphetamine-induced mania models, reducing oxidative damage and pro-inflammatory cytokine production via SIRT1/AMPK activation and Nrf2/HO-1 upregulation [31].

Luteolin has demonstrated neuroprotective effects in models of oxidative stress-induced neuronal damage relevant to BD pathophysiology. Its ability to inhibit the NF-κB/NLRP3 axis, reduce mitochondrial reactive oxygen species (ROS) production, and promote BDNF expression in hippocampal tissue, as shown in sleep deprivation and chronic pain models, positions it as a candidate for addressing neuroprogression associated with recurrent BD episodes [32,33]. Similarly, apigenin has been shown to reduce corticosterone-induced neuroinflammation and attenuate microglial activation in hippocampal regions, shifting microglia from pro-inflammatory M1 to anti-inflammatory M2 phenotypes—effects that are particularly relevant given the documented HPA axis dysregulation and hippocampal volume reduction in BD [34,35]. Epigallocatechin-3-gallate (EGCG), the major catechin in green tea, has shown protective effects against oxidative stress-induced mitochondrial dysfunction in neuronal cells, reducing caspase-3 activation and restoring mitochondrial membrane potential—mechanisms relevant to the mitochondrial impairment observed in BD [36,37].

### 3.3. Clinical Evidence and Perspectives

Clinical investigation of flavonoids in BD is still in its early stages; however, preliminary evidence supports their potential as adjunctive agents. Standard pharmacological treatments for BD (e.g., lithium, valproate, atypical antipsychotics) do not fully address the inflammatory and oxidative components of the disorder, and a significant proportion of patients show partial response or treatment-resistant BD. Therefore, there is clear justification for exploring complementary strategies [15].

N-acetylcysteine (NAC), a precursor to glutathione with antioxidant properties, has provided the most robust clinical evidence as an adjunctive treatment in BD. In a landmark randomized, double-blind, placebo-controlled trial, Berk et al. (2008) [38] demonstrated that NAC (1 g twice daily for 24 weeks) significantly reduced depressive symptoms and improved functioning in BD patients. A meta-analysis by Nery et al. (2021) [39], which included six placebo-controlled trials, confirmed a moderate effect size (d = 0.45, 95% CI: 0.06–0.84) favoring NAC over placebo for bipolar depression. While NAC is not a flavonoid, its success as an antioxidant-based adjunctive therapy in BD provides compelling proof of concept for the broader category of antioxidant and anti-inflammatory compounds, including flavonoids.

Several observational and cross-sectional studies have reported that higher dietary intake of polyphenols and flavonoid-rich foods is associated with lower severity of depressive symptoms in BD patients, better cognitive performance, and reduced levels of inflammatory biomarkers, including CRP and IL-6 [19]. Resveratrol, through its SIRT1/AMPK-dependent neuroprotective mechanisms and upregulation of BDNF, has demonstrated cognitive-enhancing effects in preclinical models [40] (Farzaei et al., 2018), though human trials in psychiatric populations remain limited and results are mixed [41].

Additionally, curcumin, a polyphenol with mechanisms overlapping several flavonoid classes, has been investigated as an adjunctive agent in mood disorders. A meta-analysis by Al-Karawi et al. (2016) [42] of six randomized trials demonstrated significant efficacy of curcumin in reducing depressive symptoms, with associated decreases in inflammatory biomarkers. A recent pilot trial in youth with treatment-resistant BD found adjunctive curcumin to be safe and tolerable, with preliminary reductions in oxidative and inflammatory markers [43]. These findings, while preliminary, are consistent with the anti-inflammatory and antioxidant mechanisms described for flavonoids and support further investigation of flavonoid-based interventions in BD.

Despite these encouraging findings, several limitations constrain the available evidence. Most clinical studies have small sample sizes and short durations, lack adequate control for dietary confounders, and do not stratify by mood state (manic, depressive, or euthymic) or illness phase. Furthermore, known interactions between flavonoids and cytochrome P450 enzymes raise important pharmacokinetic considerations in BD patients receiving polypharmacy regimens, including lithium, anticonvulsants, and antipsychotics, necessitating careful evaluation in future trials. The development of bioavailability-enhanced flavonoid formulations may be particularly important in BD, given evidence of impaired intestinal absorption and altered drug metabolism in this population [44].

The neuroinflammatory and oxidative stress profile of BD creates a compelling rationale for flavonoid-based therapeutic approaches. Although direct clinical evidence remains limited, the convergence of mechanistic data from preclinical models, promising results from related polyphenol trials, and the established anti-inflammatory and neuroprotective properties of specific flavonoids support the development of well-designed clinical trials targeting BD. Future research should focus on standardized flavonoid formulations, phase-specific interventions (manic vs. depressive episodes), integration of biomarker-guided stratification (e.g., inflammatory subgroups of BD), and careful evaluation of drug–flavonoid interactions to fully realize the therapeutic potential of these compounds in this complex and underserved disorder.

## 4. Flavonoids and Natural Compounds with Anti-Inflammatory and Neuroprotective Potential

The increasing understanding of the pathogenesis of neurodegenerative diseases and psychiatric disorders highlights the significant roles of neuroinflammation and oxidative stress [45,46]. Consequently, natural compounds, particularly flavonoids, have emerged as promising candidates due to their potent anti-inflammatory and neuroprotective properties [47,48]. To provide the necessary biochemical and pharmacological context for the preclinical and clinical evidence discussed in Section 5, this section offers a structured overview of the major flavonoid subclasses and their principal dietary sources (Section 4.1), other relevant natural compounds with overlapping neuroprotective mechanisms (Section 4.2), and the main molecular mechanisms by which these compounds affect neuroinflammation, oxidative stress, neurotransmission, and the Microbiota–Gut–Brain axis (Section 4.3). This foundational framework is intended to support the interpretation of the specific preclinical and clinical findings presented in the following sections of this review.

### 4.1. Classification and Main Sources of Flavonoids

Flavonoids are a large and diverse group of polyphenolic compounds found abundantly in fruits, vegetables, grains, tea, and wine [2,49,50]. They share a common C6-C3-C6 carbon skeleton, and their classification into subclasses depends on modifications to this structure, such as hydroxylation, methylation, and glycosylation patterns [51]. More than 10,000 flavonoid derivatives have been identified, each with distinct biological activities [51,52]. Their ability to regulate reactive oxygen species makes them important antioxidants, contributing significantly to plant stress tolerance and human health through anti-inflammatory and antimicrobial properties [53]. The major flavonoid subclasses include flavones, flavonols, isoflavones, anthocyanins (and their aglycones, anthocyanidins), flavanones, and flavan-3-ols (also known as flavanols) [2,50]. Each subclass has unique structural features and is concentrated in specific dietary sources, offering a wide range of potential health benefits [54].

#### 4.1.1. Flavones

Flavones are characterized by a double bond between carbons 2 and 3 and a ketone group at position 4 of the C ring, while in fruits and vegetables they typically have a hydroxyl group at position 5 of the A ring. The presence of hydroxyl groups at other positions, such as 7 of the A ring and 3 and 4 of the B ring, may vary depending on the taxonomic classification of the vegetable or fruit [55]. These compounds are generally found as luteolin and apigenin glycosides in leaves, flowers, and fruits, with examples including celery, parsley, red peppers, chamomile, mint, and ginkgo biloba [56]. Luteolin and apigenin are low molecular weight polyphenols present in the human diet. Although they are found in low quantities and have limited bioavailability, they are rapidly absorbed, undergo complex biotransformation processes, and are slowly eliminated, which may explain their positive biological effects on various pathologies [57]. Luteolin inhibits the activity of xanthine oxidase, which is involved in the production of ROS, while apigenin reduces the phosphorylation of NF-kB/p65 in human macrophages and monocytes, decreasing its function as a transcription factor and the production of pro-inflammatory cytokines [58].

#### 4.1.2. Flavonols

Flavonols possess a ketone group and a hydroxyl group at position 3 of the C-ring. They can be glycosylated or modified by methylation and hydroxylation and are components of proanthocyanidins. The most studied flavonols include quercetin and kaempferol, which are found in fruits and vegetables such as onions, cabbage, lettuce, tomatoes, apples, grapes, and berries [59]. Both compounds reduce the generation of ROS and promote their elimination [60]. Quercetin acts as an antioxidant and modulates intracellular signaling pathways [61]. Kaempferol is attributed with antidepressant and anxiolytic effects, related to its antioxidant and anti-inflammatory actions, activating the AKT/β-catenin pathway in the prefrontal cortex and reducing the levels of IL-1β and TNFα [62,63].

#### 4.1.3. Isoflavones

Isoflavones, which have a phenyl group attached to the third carbon of the benzopyran ring, are especially abundant in legumes, particularly soybeans and soy-derived products [64]. Genistein and daidzein are major isoflavones recognized for their phytoestrogenic activity and various health benefits [65].

#### 4.1.4. Anthocyanins

They are a group of red pigments that vary depending on pH and chemical modifications, such as methylation or acylation, that occur in the hydroxyl groups of the A and B rings. They act as pigments responsible for the coloration of various plants and fruits, including blueberries, currants, grapes, raspberries, strawberries, and blackberries. Among the most widely studied anthocyanins are cyanidin and malvidin [66]. Cyanidin extracts have been shown to have antidepressant effects, as they can increase the levels of monoaminergic neurotransmitters such as NE and 5-HT through various mechanisms. These include inhibition of the enzyme monoamine oxidase (MAO) and stimulation of the production of BDNF. These effects are related to the promotion of neurogenesis and the development of dendrites in the hippocampus, which contributes to reversing depressive behaviors. Malvidin, found primarily in red wine, has been shown to have neuroprotective properties against oxidative damage [67].

### 4.2. Other Natural Compounds with Anti-Inflammatory and Neuroprotective Potential

Beyond flavonoids, a wide range of other natural compounds exhibit significant anti-inflammatory and neuroprotective activities, often with overlapping mechanisms and synergistic effects. These include various polyphenols, phenolic acids, terpenoids, and alkaloids [47].

#### 4.2.1. Polyphenols

Polyphenols are secondary plant metabolites found in fruits, vegetables, olive oil, chocolate, tea, and red wine. Their production is activated by external factors such as heat, UV radiation, pests, or infections. Due to their hydroxyl groups, they act as antioxidants, neutralizing free radicals and protecting tissues. They also reduce oxidative stress and neuronal inflammation, helping protect neurons and improve cognitive functions such as memory and learning [68].

Resveratrol (3,4′,5-trihydroxystilbene) is a phenolic stilbene with preventive and therapeutic effects against cardiovascular and inflammatory diseases, diabetes, cancer, infections, and neurodegenerative disorders [69]. It exists in two isomers, cis and trans, with the trans isomer being the most active [70]. Resveratrol also exerts neuroprotective effects by reducing depressive and anxiety behaviors through inhibition of MAO increasing 5-HT and DA, activating the BDNF/TrkB pathway, and modulating the HPA axis to decrease cortisol. Additionally, it has anti-inflammatory effects by inhibiting NF-κB and NLRP3, contributing to the reduction in neuroinflammation associated with depression [71,72]. Curcumin (1,7-bis(4-hydroxy-3-methoxyphenyl)-1,6-heptadiene-2,5-dione) has anti-inflammatory, antioxidant, anticancer, hepatoprotective, and cardioprotective properties, as well as effects against hypoglycemia, arrhythmias, and neurological disorders [73]. It also acts by eliminating reactive oxygen species, stimulating glutathione, and modulating serotonergic and dopaminergic neurotransmission by inhibiting monoamine oxidase and modulating the HPA axis, which supports its anxiolytic and antidepressant effects [74].

#### 4.2.2. Phenolic Acids

Phenolic acids are natural compounds found in fruits and vegetables, classified as derivatives of hydroxybenzoic and hydroxycinnamic acids. Hydroxybenzoic acids contain a carboxylic group, while hydroxycinnamic acids have the structure C6H5CH=CHCOOH, which can be modified with hydroxyl groups. This group includes compounds such as p-hydroxycinnamic, p-coumaric, rosmarinic, caffeic, and ferulic acids [75]. These compounds have various biological functions, including antihypertensive, anticancer, anti-inflammatory, neuroprotective, and antidepressant effects [76]. Rosmarinic acid, found in Lamiaceae plants, exerts anxiolytic and antidepressant effects by modulating the endocannabinoid system (CB_1_ and CB_2_), the PPAR-γ receptor, and the Nrf2 antioxidant pathway. It also reduces depressive-like behaviors induced by inflammation and oxidative stress and enhances the activity of antioxidant enzymes such as superoxide dismutase (SOD), catalase (CAT), and enzymes involved in glutathione synthesis (GSH) [77,78]. Caffeic acid, a hydroxycinnamic acid present in fruits, vegetables, grains, herbs, and coffee, has antioxidant, anti-inflammatory, and neuroprotective properties. Some studies report anxiolytic and antidepressant effects through modulation of pathways related to oxidative stress, inflammation, and neuroplasticity, improving depressive-like behaviors, reducing pro-inflammatory cytokines, and restoring BDNF levels [79,80,81].

#### 4.2.3. Terpenoids and Alkaloids with Neuroprotective Activity

Terpenoids such as β-caryophyllene, limonene, linalool, 1,8-cineole, and eugenol exhibit anxiolytic, antidepressant, and neuroprotective effects. These compounds restore BBB permeability, inhibit neuroinflammation through the PI3K/Akt [82] and NF-κB/NLRP3 pathways, reduce oxidative stress, and promote BDNF expression, supporting neuroplasticity and neuronal survival [83]. Their lipophilicity and low molecular weight enable them to cross the BBB and modulate neurotransmitters and inflammatory pathways in the CNS, making them useful as complementary treatments for anxiety and depression [84]. Some natural alkaloids, such as harmaline and mesembrine, also show neuroprotective effects; harmaline increases BDNF and modulates serotonergic 5-HT_2A_ receptors, while mesembrine inhibits serotonin reuptake, promoting neuronal plasticity. Other alkaloids, such as matrine, reduce neuroinflammation and oxidative stress, producing anxiolytic and antidepressant effects [85].

### 4.3. Mechanisms of Action of Flavonoids and Natural Compounds

**Figure 1:**
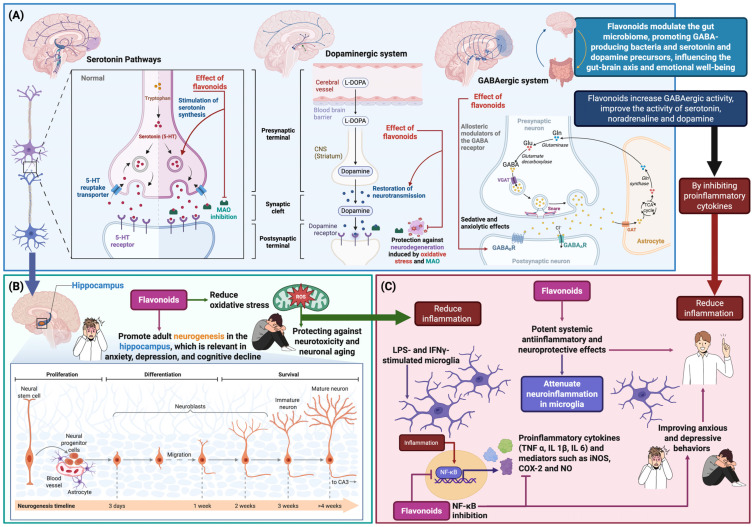
Mechanisms of action and therapeutic effects of flavonoids. (**A**) Interaction with neurotransmitters. (**B**) Regulation of oxidative Stress and increased neurogenesis. (**C**) Anti-inflammatory effect of flavonoids. Description in the text. Figure created with Biorender. Muñoz-Carrillo, J.L. (2026) https://www.biorender.com (accessed on 20 March 2026).

Flavonoids have multiple therapeutic effects by modulating neurotransmitters such as gamma-aminobutyric acid (GABA), 5-HT, DA, and NE, regulating the HPA axis, enhancing BDNF/TrkB signaling, reducing neuroinflammation and oxidative stress, and controlling endoplasmic reticulum stress, making them promising compounds for anxiety, depression, and neuronal deterioration (Figure 1) [67]. Compounds such as quercetin, luteolin, apigenin, chrysin, baicalin, naringenin, and icariin modulate GABA_A_, 5-HT_1A_, 5-HT_2A_, α-adrenergic, and D1/D2 receptors, inhibit MAO, and activate TrkB, thereby stabilizing neurotransmitters and promoting neurogenesis in the hippocampus [48,86]. Furthermore, flavonoids reduce oxidative stress and inflammation by inhibiting ROS, pro-inflammatory cytokines (TNF-α, IL-1β, IL-6), cyclooxygenase-2 (COX-2), and xanthine oxidase, and by activating antioxidant pathways such as Nrf2-ARE, thus protecting against neuronal damage and microglial activation. Regarding endoplasmic reticulum stress, compounds such as wogonin, luteolin, and rutin regulate protein folding by activating caspases, alpha subunit of eukaryotic initiation factor 2 (eIF2α), glucose-regulated protein 78 (GRP78), and autophagy or controlled apoptosis pathways, preventing the accumulation of misfolded proteins in neurodegenerative diseases [87].

#### 4.3.1. Inhibition of NF-κB and Reduction in Inflammatory Cytokines

Flavonoids exhibit potent systemic anti-inflammatory and neuroprotective effects, primarily by inhibiting NF-κB, which reduces TNF-α, IL-1β, IL-6 and mediators such as inducible nitric oxide synthase (iNOS), COX-2, and nitric oxide (NO). For example, resveratrol blocks NF-κB activation in lipopolysaccharides (LPS) and interferon gamma (IFN-γ)-stimulated microglia, decreasing TNF-α and IL-1β and improving anxious and depressive behaviors through the NF-κB/NLRP3 pathway. Flavonoids such as naringenin inhibit microglial activation (BV2), reduce iNOS, TNF-α, and IL-1β, and suppress MAPK phosphorylation (JNK, ERK, p38) [88]. Other relevant phytochemicals include baicalein from *Scutellaria baicalensis*, which suppresses TNF-α-induced NF-κB and decreases the expression of related genes; the sesquiterpene lactone parthenolide, which inhibits NF-κB and reduces TNF-α, IL-6, and IL-17 in brain injury models; and thymoquinone from *Nigella sativa*, which attenuates neuroinflammation in BV2 microglia by suppressing NF-κB and activating Nrf2/SIRT1/AMPK, reducing TNF-α, IL-6, PGE2, and NO, and demonstrating neuroprotection in Alzheimer’s models [89]. Furthermore, Panax ginseng ginsenosides (Re and Rg6) inhibit IKKβ phosphorylation and NF-κB activation, block TLR4/IRAK signaling, and decrease TNF-α and IL-1β in macrophages stimulated with LPS or experimental colitis [90]. A recent meta-analysis indicates that modulators of the TLR2/4MyD88 NF-κB pathway reduce IL-1β, IL-6, and TNF-α, while increasing anti-inflammatory cytokines such as IL-4 and IL-10, exerting neuroprotective effects in chronic neurological disorders [91].

#### 4.3.2. Regulation of Oxidative Stress and Increased Neurogenesis

Flavonoids, polyphenols, and isothiocyanates can reduce oxidative stress and promote adult neurogenesis in the hippocampus, which is relevant to anxiety, depression, and cognitive impairment [92]. Flavonoids neutralize ROS and donate protons through their hydroxyl groups, inhibiting lipid and protein oxidation, and increasing GSH and antioxidant enzymes (SOD, CAT, GPx) via the Nrf2 ARE pathway, protecting against neurotoxicity and neuronal aging [93]. Compounds such as baicalein and naringenin restore neural proliferation and BDNF expression in the dentate gyrus of the hippocampus [94]. Resveratrol activates SIRT1/AMPK and Nrf2/HO-1, decreases oxidative stress markers, improves spatial memory, and promotes neurogenesis by increasing BDNF and CREB levels [95]. EGCG from green tea promotes neurogenesis in inflammatory models by reducing pro-inflammatory cytokines via TLR4/NF-κB, increasing dendritic precursor cells (DCX), and activating Akt [96]. 7,8-Dihydroxyflavone acts as a TrkB agonist and antioxidant, protecting against excitotoxicity and promoting synaptic plasticity and neurogenesis [95]. Other compounds, such as sulforaphane, ginsenoside Rd, and astragaloside IV, improve redox homeostasis, increase antioxidant enzymes, and reduce pro-inflammatory cytokines, promoting BDNF-mediated neurogenesis [93,94].

#### 4.3.3. Interaction with Neurotransmitters

Flavonoids, alkaloids, and terpenoids directly modulate neurotransmitter systems involved in mood, anxiety, and depression. In the serotonergic system, substances such as naringenin, icariin, and quercetin increase 5-HT levels in the hippocampus and other regions by stimulating its synthesis or inhibiting its degradation by MAO [67,97]. In the dopaminergic system, compounds such as catechin, quercetin, and icariin restore neurotransmission and protect against neurodegeneration caused by oxidative stress and MAO [97,98]. In the GABAergic system, flavonoids such as apigenin and amentoflavone act as allosteric modulators of GABA_A_ receptors, promoting sedative and anxiolytic effects, while passionflower and the alkaloid leonurine increase GABAergic activity and improve 5-HT, NE, and DA levels by inhibiting pro-inflammatory cytokines [97,99]. Hyperforin, found in *Hypericum perforatum*, inhibits the reuptake of 5-HT, NE, DA, and GABA, increasing their synaptic concentrations and improving mood [100]. Additionally, flavonoids modulate the gut microbiome, promoting GABA-producing bacteria and the precursors of 5-HT and DA, thereby influencing the gut–brain axis and emotional well-being [101]. Figure 1 describes the mechanisms of action and therapeutic effects of flavonoids.

#### 4.3.4. Modulation of the Microbiota–Gut–Brain Axis

The MGB axis is a bidirectional communication system linking the gut microbiome with the CNS. This axis plays a significant role in influencing brain function, mood, and behavior, and its dysregulation is implicated in various neurological and psychiatric conditions [97,102]. Flavonoids and their metabolites can modulate the composition and function of the gut microbiota, which in turn influences the production of neuroactive compounds and SCFAs that affect brain health [102]. The bioconversion of dietary flavonoids by gut microbes generates therapeutic metabolites that can be transported to the brain, influencing inflammation and oxidative stress [102]. Butyrate activates AMPK/PGC1α in neuronal mitochondria, enhances autophagy, decreases oxidative stress, and promotes mitochondrial biogenesis, protecting brain function and reducing depressive symptoms [103]. Strains such as *Lactobacillus rhamnosus* JB-1 produce intestinal GABA that communicates signals to the brain via the vagus nerve, modulating GABAA and GABAB receptors and decreasing stress-induced HPA axis activation [97]. Studies have observed that prebiotics such as FOS and GOS favor *Bifidobacterium longum*, reduce HPA axis activation, increase brain BDNF, and alleviate anxiety and depression, while synbiotic combinations showed significant increases in BDNF and better clinical outcomes than probiotics alone [101,104,105]. This interaction highlights a sophisticated mechanism through which natural compounds, even those with limited direct bioavailability, can exert significant neuroprotective and anti-inflammatory effects by reshaping the gut environment and its communication with the brain [12,97].

## 5. Preclinical and Clinical Evidence on the Effect of Flavonoids on Neuroinflammation and Affective Disorders

Neuroinflammation has emerged as a critical pathophysiological mechanism underlying various neuropsychiatric disorders, including anxiety and depression. Activation of microglia and astrocytes, along with increased production of TNF-α, IL-1β, and IL-6, disrupts normal neurobiological processes and contributes to mood disorders [46]. Flavonoids, a diverse class of polyphenolic compounds found in fruits, vegetables, and medicinal plants, have demonstrated significant neuroprotective properties by modulating neuroinflammatory pathways. Preclinical evidence increasingly supports the therapeutic potential of these natural compounds in reducing inflammatory markers and reversing behavioral deficits associated with anxiety and depression [3,9,106].

### 5.1. Animal Models of Anxiety and Depression: Effects of Flavonoids on Reducing Neuroinflammation and Improving Behavior

Preclinical studies primarily use rodent models to replicate aspects of human affective disorders and investigate potential therapeutic interventions. These models, including chronic unpredictable mild stress (CUMS), forced swim test (FST), tail suspension test (TST), and various anxiety paradigms, reliably induce behavioral changes such as anhedonia, despair, and increased anxiety-like behaviors, as well as neuroinflammatory responses that can be modulated by antidepressant and anxiolytic compounds [86,107]. Flavonoids have consistently shown beneficial effects in these models, improving behavioral phenotypes and alleviating neuroinflammatory markers.

Quercetin is one of the most extensively studied flavonoids in the context of neuroinflammation and mood disorders (see Section 4.1.2 for structural and dietary context). Multiple preclinical studies have demonstrated its ability to reduce inflammatory responses and improve behavioral outcomes in animal models. In an LPS-induced depression model, administration of quercetin (50 mg/kg orally for seven days) significantly reduced depressive-like behaviors in rats, as shown by improved performance in the FST and increased sucrose preference test (SPT) scores [29]. Mechanistically, quercetin treatment significantly reduced TNF-α, IL-6, and IL-17 in brain tissue, while also suppressing the expression of NF-κB, NLRP3 inflammasome, and inducible iNOS in the hippocampus and prefrontal cortex [29].

In anxiety models, quercetin (100 mg/kg daily for 21 days) significantly improved anxiety-like behaviors in rats with LPS-induced neuroinflammation [108]. The treatment reduced hippocampal levels of IL-6, IL-1β, COX-2, and NF-κB while enhancing BDNF expression, demonstrating that the anxiolytic effects were directly linked to suppression of neuroinflammatory cascades [108]. Quercetin also attenuated astrocyte activation and reduced IL-1β and TNF-α levels in a methamphetamine-induced anxiety model, while improving mitochondrial dysfunction [109]. Beyond its behavioral effects, quercetin ameliorates cognitive impairment in depression by targeting heat shock protein 90 (HSP90) to inhibit NLRP3 inflammasome activation in microglia, consistent with its broader anti-inflammatory mechanisms described in Section 4.3.1. This treatment reduced cytokine levels (IL-6, IL-1β, monocyte chemoattractant protein-1, and TNF-α) in the hippocampus and reversed depression-like behavior and cognitive deficits in CUMS [110].

Luteolin has demonstrated potent anxiolytic and antidepressant properties through multiple anti-inflammatory mechanisms (see Section 4.1.1 for structural and dietary context). In a single prolonged stress (SPS) model of post-traumatic stress disorder, luteolin administration (10 and 20 mg/kg daily for 14 days) significantly reduced fear freezing responses, depression-like behaviors, and anxiety-like symptoms in rats [111]. These behavioral improvements were accompanied by suppression of plasma corticosterone and adrenocorticotropic hormone levels, normalization of HPA axis function, and restoration of 5-HT and NE levels in the prefrontal cortex and hippocampus. Luteolin’s modulation of neuroinflammation involves downregulation of key inflammatory pathways, consistent with the NF-κB/NLRP3 inhibitory mechanisms described in Section 4.3.1. In sleep deprivation-induced anxiety and depression models, luteolin treatment (10 and 20 mg/kg for 21 days) effectively ameliorated behavioral alterations by suppressing the NF-κB/NLRP3 inflammasome axis in the hippocampus [32]. Molecular analysis showed downregulation of NF-κB, NLRP3, apoptosis-associated speck-like protein containing a CARD (ASC), and active caspase-1, indicating comprehensive inhibition of the inflammasome-mediated inflammatory cascade.

Recent investigations have shown that luteolin’s antidepressant effects involve modulation of microglial phenotype. In chronic stress models, luteolin administration promoted the arginase-1+ microglial phenotype through a peroxisome proliferator-activated receptor gamma (PPARγ)-dependent mechanism, reducing hippocampal inflammation and ameliorating depressive-like behaviors [112]. Additionally, luteolin treatment significantly attenuated LPS-induced neuroinflammation by decreasing IL-6 production in brain-derived astrocytes and reducing serum levels of IL-6, TNF-α, and corticosterone, while increasing mature BDNF, DA, and NE levels in the hypothalamus [113]. In chronic neuropathic pain-induced mood disorders, luteolin (10, 25, and 50 mg/kg for 21 days) significantly reduced anxiety and depression-like behaviors and decreased neuroinflammatory markers in the hippocampus and prefrontal cortex [33]. The treatment increased levels of BDNF, glial cell-derived neurotrophic factor (GDNF), and antioxidant enzymes, demonstrating that behavioral improvements were mediated through both anti-inflammatory and neuroprotective mechanisms.

For example, the flavonoid pinocembrin, isolated from honey and propolis, has been shown to mitigate depressive-like behaviors induced by CUMS in mice [114]. Animals subjected to CUMS typically show reduced sucrose preference, increased immobility in the FST and TST, and decreased locomotor activity in the open field test (OFT)—all indicators of depressive-like states. Pinocembrin treatment effectively reversed these behavioral deficits, suggesting a significant antidepressant-like effect [114]. Notably, these behavioral improvements were associated with reduced neuroinflammation, highlighting a direct link between the flavonoid’s anti-inflammatory action and its mood-modulating effects [114].

Similarly, chrysin, a flavonoid found in plants such as *Passiflora coerulea*, has been extensively investigated for its anxiolytic and antidepressant-like properties in preclinical studies (see Section 4.1.1 for structural and dietary context). Research indicates that chrysin exerts its beneficial effects by modulating specific neurotransmitter systems, primarily GABAergic and serotonergic pathways, which are critical for regulating anxiety and depression [48,106,115]. In addition to these neurochemical interactions, chrysin’s anti-inflammatory and antioxidant activities are considered crucial mechanisms contributing to its anxiolytic and antidepressant effects, particularly in pathophysiological contexts involving neuroinflammation and apoptosis [106]. The consistent demonstration of chrysin’s therapeutic potential in preclinical research suggests a promising role as an anxiolytic and antidepressant agent [106]. Table 1 describes the effects of flavonoids on neuroinflammation and affective disorders in animal models.

Flavonols, a prominent subclass of flavonoids, have also shown significant promise as antidepressant agents in preclinical models. They can restore neuroendocrine control, promote neurogenesis, and alleviate depressive-like behaviors, effects largely attributed to their potent antioxidative and anti-inflammatory properties [125]. Evidence from animal studies strongly supports the idea that flavonoids can improve behavioral outcomes in models of anxiety and depression by addressing underlying neuroinflammatory processes. Specific flavonoids have been shown to prevent anxiety-like behaviors and dysregulation of cytokine systems. For example, cyanidin, an anthocyanin flavonoid, was found to prevent anxiety-like behaviors and cytokine dysregulation during abstinence from synthetic cathinone in rats. This demonstrates the ability of flavonoids to intervene in stress-induced neuroinflammatory processes that contribute to affective disorders [126].

### 5.2. Impact of Flavonoids on the Modulation of Inflammation in Humans León

Scientific evidence has shown that inflammation underlies several diseases, particularly metabolic, neurodegenerative, autoimmune, and cardiovascular diseases [127]. Flavonoids, whose classification, dietary sources, and primary anti-inflammatory mechanisms are described in Section 4.1 and Section 4.3, have been the focus of numerous clinical studies, especially due to their documented ability to decrease pro-inflammatory cytokine production, reduce oxidative stress, and modulate immune-related signaling pathways [128]. The following subsection summarizes the available clinical evidence on the anti-inflammatory effects of flavonoids in human populations with various inflammation-related conditions. This has enabled scientists to assess the in vivo impact of these molecules by using patients with a spectrum of inflammation-related diseases.

Firstly, the more extensive publications that connect flavonoids with anti-inflammatory action are a part of nutritional research. For instance, one trial assessed the effect of Mediterranean diet (for 12 weeks) in type 2 diabetes mellitus participants. It was observed that the former-diet promoted an elevation of naringenin, hesperetin and hesperidin plasma levels in parallel to a decrease in plasma pro-inflammatory cytokines interleukin-6 (IL-6) expression, this was not detected with a routine diet. This therefore indicates that such citrus flavonoids present in the Mediterranean diet also may have possibly resolved and ameliorated the inflammatory status of diabetic mellitus individuals [129].

Similarly, the consumption of concord grape juice, which is rich in flavonoids, improved the inflammatory profile, including the reduction in C-reactive protein (CRP) in healthy smokers [130]. Furthermore, isoflavone consumption (34 mg daily administered for 56 days) decreased Th1-type cytokines and markers associated with myeloid-derived suppressor cells (MDSC), in addition to increasing natural killer (NK) cells and reducing the percentage of regulatory T-cells (Treg), suggesting a regulatory role on the immune system in patients with prostate cancer [131].

In another aspect, the anti-inflammatory effects have not only been demonstrated for flavonoid-rich diets through clinical trials, but also for vegetable sources derived from fruits containing flavonoids. Such is the case with purple raspberries, which are rich in anthocyanins and berberine. These have been shown to decrease CRP levels and improve the lipid profile in individuals at cardiovascular risk after being administered for 8 weeks (10 g per day) [132].

On the other hand, obesity is one of the most prevalent diseases worldwide and has a significant association with inflammatory processes [133]. In this context, the properties of some flavonoids mainly citrus flavonoids such as tangeretin, sinensetin, and nobiletin have shown anti-inflammatory effects in people with obesity. For example, in a randomized, double-blind, and placebo-controlled study, the daily consumption of flavonoid-enriched orange juice (200 mL) for six weeks, administered alongside a hypocaloric diet, decreased low-density lipoprotein (LDL) levels, increased glutathione peroxidase 1 activity, and decreased IFN-γ and TNF-α, showing a clear regulation of the inflammatory processes implicated in obesity [134].

Moreover, in neurodegenerative conditions, some flavonoids such as anthocyanidins have been shown to possess the capacity to neutralize free radicals and inhibit pro-inflammatory genes (see Section 4.1.4 for structural and dietary context). Consistent with this, a study by Pedret et al. (2024) [135] reported that supplementation with a diet enriched with apple-derived anthocyanins reduced CRP and IL-6 in six weeks. However, the anti-inflammatory properties of anthocyanidins lack recent or entirely clear studies, as another 24-week clinical trial did not observe significant changes in inflammatory biomarkers despite the cognitive improvement induced by anthocyanin supplementation [136].

In specific cases, some research has focused on evaluating isolated flavonoids such as rutin, which in one study was administered for three months in people with type 2 diabetes mellitus. It showed an effect of reducing levels of low-density lipoprotein (LDL), very-low-density lipoprotein (VLDL), interleukin 6 (IL-6), and insulin, as well as increasing BDNF levels, suggesting a mechanism by which rutin modulates inflammatory, metabolic, and neuroprotective processes [137].

Another studied flavonoid is quercetin, which has shown varied results. For example, in men undergoing coronary bypass, quercetin reduced CRP while in women it showed pro-inflammatory effects [138]. Similarly, this flavonoid has been shown to reduce ultra-sensitive C-reactive protein (us-CRP) levels in healthy adults [139], while in women with rheumatoid arthritis, treatment with quercetin (500 mg/day for 8 weeks) decreased plasma levels of TNF-α, suggesting a clearer benefit in autoimmune contexts.

Other specific flavonoids that have shown anti-inflammatory activity are isoflavones like genistein, a widely studied soy isoflavone. In this regard, in a study by Amanat et al. (2018) [140], genistein reduced the levels of TNF-α and IL-6, accompanied by an improvement in insulin resistance, oxidative stress, and lipid metabolism in patients with fatty liver after eight weeks of oral supplementation.

Another studied flavonoid is hesperidin, whose administration of 500 mg/day for six weeks promoted a reduction in CRP and IL-6 and systolic blood pressure in patients with type 2 diabetes mellitus, indicating anti-inflammatory and antihypertensive effects [141]. Likewise, baicalin also demonstrated benefits by decreasing triglycerides and CRP in patients with coronary artery disease and rheumatoid arthritis after 12 weeks of treatment [142].

Finally, the most recent and available scientific evidence demonstrates that flavonoids exert meaningful regulatory effects on inflammatory processes in humans across a range of clinical conditions. Critically, the inflammatory biomarkers shown to be reduced by flavonoids in these studies—including TNF-α, IL-1β, IL-6, and CRP—are the same markers consistently elevated in patients with anxiety, depression, and BD, as described in Section 3 and Section 5.3. Furthermore, the clinical populations studied in this section, including patients with type 2 diabetes mellitus, obesity, and cardiovascular disease, present substantially elevated rates of comorbid affective disorders, making the anti-inflammatory effects of flavonoids documented in these contexts directly relevant to the management of neuroinflammation-driven mood disturbances. These findings therefore provide essential translational evidence supporting the biological plausibility of flavonoid-based interventions in affective disorder populations, complementing the more specific clinical evidence presented in Section 5.3.

### 5.3. Clinical Effects of Flavonoids on Anxiety and Depressive Symptoms Associated with Inflammatory Regulation

Anxiety and depression have been closely linked to the development of systemic and cerebral inflammatory processes, as well as to oxidative stress and alterations in the gut microbiota [143,144,145]. In this context, flavonoids have been proposed as potential anxiolytic and antidepressant alternatives. These compounds have shown such effects at the preclinical level through various mechanisms, including antioxidant and neuroprotective actions, increased levels of neurotransmitters (such as 5-HT and DA) and neurotrophic factors (such as BDNF and Neuronal Growth Factor [NGF]), and, more recently, anti-inflammatory properties [83]. However, clinical evidence remains limited. Nevertheless, available studies have identified beneficial effects on symptoms of depression and anxiety in patients with or without conditions associated with inflammatory processes—areas of research that are still evolving.

For example, in a study of patients with multiple sclerosis, daily administration of crocin (30 mg for eight weeks) reduced anxiety symptoms and CRP levels, suggesting an emotional benefit associated with inflammatory modulation [146]. Another study with the flavolignan silybin (94 mg/day for 12 months) significantly decreased symptoms of depression and anxiety in patients with hepatitis C, in addition to reducing hepatic enzymes ALT and AST, suggesting an indirect improvement in emotional status through decreased liver damage and systemic inflammation [147]. Similarly, some studies have explored flavonoid-enriched supplementation for symptoms of anxiety and depression. For instance, in a clinical trial involving young adults with depressive symptoms, consumption of flavonoid-rich orange juice (approximately 600 mg/day) for eight weeks improved affective symptoms, increased BDNF levels, reduced CRP levels, and promoted the proliferation of anti-inflammatory gut bacteria, suggesting a relationship between inflammatory regulation, the microbiota, and emotional well-being [148].

Other studies have examined combined interventions, such as in multiple sclerosis patients treated for four months with EGCG (800 mg) alongside coconut oil. In this study, both groups showed decreases in anxiety symptoms and IL-6 levels, making it difficult to attribute the anxiolytic effect exclusively to flavonoids [149]. In addition to the limited clinical information on the anxiolytic or antidepressant effects of flavonoids with anti-inflammatory properties, some studies report inconclusive results. For example, recent research found that administering quercetin to post-infarction patients did not change depression symptoms or levels of intercellular adhesion molecule 1 (ICAM-1) and vascular adhesion molecule 1 (VCAM-1). The authors suggest that this lack of significant changes could be due to participants not having clinical depressive symptoms or elevated inflammation at the start of the study [150].

Another example is a study in healthy older adults, where eight weeks of purple potato anthocyanin supplementation improved symptoms of depression, irritability, and stress; however, it did not significantly change the marker 8-hydroxy-2′-deoxyguanosine (8-OHdG), an indicator of oxidative DNA damage, preventing confirmation of the proposed anti-inflammatory action [151]. Similarly, in young people with major depression, supplementation with a flavonoid-rich diet for eight weeks did not significantly change LPS levels, although a downward trend was observed compared to the low-flavonoid diet group [152]. Finally, in adults with type 2 diabetes mellitus and depressive symptoms, daily consumption of 700 mg of hydroalcoholic extract for 12 weeks reduced anxiety and depression, but did not change inflammatory markers such as CRP, suggesting the involvement of mechanisms other than immunomodulation [153].

Overall, the evidence shows inconclusive results regarding the anxiolytic and antidepressant effects associated with anti-inflammatory properties in clinical settings, as the number of studies supporting this relationship matches those showing no clear effects. Therefore, it is not possible to conclusively establish that these effects are specifically due to modulation of inflammatory pathways. Nevertheless, the evidence remains limited, despite numerous preclinical studies associating these actions on affective state with regulation of inflammatory processes in animal models. Further clinical research is needed to clarify the mechanisms involved in the effects of flavonoids on mental health.

### 5.4. Challenges in Clinical Research of Flavonoids in Anxiety and Depression Associated with Inflammation

Based on previous information about the anti-inflammatory effects of flavonoids and their relationship with anxiety and depression in clinical research, it is clear that few studies directly address this correlation. Although multiple preclinical studies have shown that flavonoids modulate pro-inflammatory cytokines, oxidative stress, and various neuroinflammatory pathways, attempts to replicate these findings in humans have produced inconclusive results. This may be due to ethical limitations and the lack of simultaneous measurement of inflammatory markers and affective symptoms, even though this interaction is fundamental to understanding the mechanisms involved in the effects of these substances.

Another important limitation is the inconsistency of clinical results. Although several studies have shown that flavonoids whether isolated, co-administered, or as part of diets rich in these metabolites can improve symptoms of anxiety and depression in healthy individuals or those with a clinical condition, the anti-inflammatory properties do not always accompany these beneficial effects on mental health. This disconnect makes it difficult to establish a causal association between the anxiolytic or antidepressant effect and the modulation of inflammatory pathways.

Finally, another challenge in the available evidence is the wide methodological variability among clinical trials. These differences include the route of administration, the dose of flavonoids, the form in which they are presented whether in diets rich in these metabolites, as isolated flavonoids, or as extracts as well as the duration of treatment and the characteristics of the populations studied, which may or may not have a prior diagnosis of anxiety or depression. These variations limit the ability to consistently detect an anxiolytic, antidepressant, or anti-inflammatory effect. Additionally, the presence or absence of inflammation in participants (for example, in conditions such as diabetes, multiple sclerosis, hepatitis C, obesity, or major depression) further complicates comparisons between studies and reduces the ability to draw general conclusions.

## 6. Therapeutic Applications and Future Perspectives

### 6.1. Optimized Formulations and Enhanced Bioavailability

As described in Section 5.1, preclinical evidence has demonstrated superior anxiolytic and antidepressant efficacy for nanotechnology-based flavonoid formulations, including intranasally administered quercetin-loaded polymeric nanocapsules and BDNF-quercetin alginate nanogels, compared to conventional oral administration. Building on this preclinical foundation, this section addresses the broader translational imperatives and pharmacokinetic strategies that must be pursued to optimize the clinical application of flavonoids in affective disorders.

Despite their broad neurotherapeutic potential, the clinical application of flavonoids in affective disorders is critically limited by poor oral bioavailability, which results from low solubility, rapid metabolism, and limited BBB penetration [104]. To address these pharmacokinetic barriers, nanotechnology-based delivery systems including polymeric nanoparticles, liposomes, and nanogels have emerged as advanced strategies to optimize drug disposition and targeting [104,154]. For example, quercetin-loaded polymeric nanocapsules administered intranasally demonstrate superior anxiolytic efficacy compared to oral quercetin dispersion, with improved BBB permeability and behavioral outcomes in preclinical anxiety models [154]. Similarly, nanocurcumin formulations show markedly enhanced bioavailability and antidepressant activity. Clinical meta-analyses comparing conventional and nanoenhanced delivery highlight this point: nanocurcumin produced substantial benefits in reducing depressive symptoms (SMD = −1.30, 95% CI: −2.31 to −0.30), whereas conventional curcumin formulations showed negligible impact (SMD = −0.40, 95% CI: −1.04 to 0.24), underscoring the critical role of bioavailability enhancement [155].

Dual-drug nanocarrier systems co-loading flavonoids with SSRIs or other psychotropic agents represent another promising frontier, demonstrating synergistic neuroplastic and anti-inflammatory effects through concurrent modulation of NF-κB, BDNF, and monoaminergic pathways [104]. Stimuli-responsive nanoplatforms activated by pH gradients or enzymatic cues enable targeted, sustained release in neuroinflammatory brain regions [156]. Despite these innovations, challenges related to manufacturing scalability, cost-effectiveness, and regulatory approval remain substantial barriers to clinical implementation [104].

### 6.2. Flavonoids as Adjunctive Therapy

The preclinical and clinical evidence synthesized in Section 5 collectively supports the potential of specific flavonoids—particularly quercetin, luteolin, apigenin, and chrysin—as adjunctive agents in the pharmacological management of affective disorders. Building on this evidence base, this section discusses the strategic framework and mechanistic rationale for developing flavonoid-based adjunctive therapies in combination with conventional antidepressant and anxiolytic treatments.

Flavonoids show considerable potential as adjunctive agents in antidepressant therapy due to their multi-target mechanisms, including antioxidant, anti-inflammatory, and neurotrophic effects [63,116,157]. Apigenin binds to BDNF and enhances its neurotrophic activities by increasing TrkB receptor phosphorylation, thereby promoting neuroplasticity and neuroprotection [63]. In chronic stress models, apigenin reverses depressive-like behaviors by upregulating hippocampal BDNF expression and modulating CREB signaling [157]. Luteolin exhibits robust antidepressant effects by inhibiting 5-HT reuptake, reducing MAO-A activity, and upregulating BDNF expression [158].

Additionally, luteolin suppresses neuroinflammation by inhibiting the NF-kappaB/NLRP3 axis and reducing IL-1β, IL-6, and TNF-α [159]. Quercetin demonstrates dose-dependent anxiolytic and antidepressant effects through modulation of GABAergic, serotonergic, and glutamatergic systems. A meta-analysis of 52 animal studies confirmed that quercetin significantly reduces immobility time in FST, increases sucrose preference, and elevates BDNF levels while decreasing inflammatory markers [160]. These findings demonstrate that the therapeutic efficacy of flavonoids is time-dependent and directly linked to optimized bioavailability formulations. For example, curcumin interventions exceeding 8 weeks yield substantial antidepressant effects (SMD = −2.58, 95% CI: −4.54 to −0.61), whereas shorter durations show negligible benefits [161]. This positions bioavailability-enhanced flavonoids as effective adjunctive agents to conventional psychotropic drugs.

### 6.3. Nutritional Psychiatry and Dietary Interventions

The clinical evidence reviewed in Section 5.3, including findings from randomized controlled trials on flavonoid-rich dietary interventions and isolated flavonoid supplementation, provides a growing empirical foundation for the emerging field of Nutritional Psychiatry. This section discusses the broader implications of flavonoid-rich dietary patterns—including the Mediterranean diet and targeted supplementation with berry-derived anthocyanins—as accessible, sustainable, and evidence-based strategies for the prevention and adjunctive management of affective disorders at the population level [162,163]. Epidemiological studies show that individuals in the highest quintile of anthocyanidin intake have a 40% lower incidence of depression compared to those in the lowest quintile [51]. Berry consumption, which is rich in anthocyanins, shows significant promise for adolescents. A randomized, double-masked, placebo-controlled trial found that four weeks of daily wild blueberry supplementation (253 mg anthocyanins) significantly reduced depressive symptoms in adolescents (*p* = 0.02) [164]. The Mediterranean Diet, characterized by high intake of flavonoid-rich fruits, vegetables, and olive oil, is associated with lower depression risk and reduced symptom severity [165]. A systematic review found that higher polyphenol consumption is associated with a 32% reduction in depression risk [162]. Probiotic–flavonoid combinations represent a synergistic approach targeting the MGB axis, and preclinical studies have shown enhanced gut microbiota diversity, increased SCFAs production, and reduced systemic inflammation, thereby attenuating depressive-like behaviors [166].

### 6.4. Translational Imperatives and Precision Roadmaps

The clinical translation of flavonoid-based therapeutics requires large-scale, randomized controlled trials with standardized dosing, bioavailability-enhanced formulations, and validated neuropsychiatric outcomes [161,167]. The integration of systems pharmacology, omics technologies, and nanomedicine offers transformative potential for developing next-generation flavonoid therapeutics with improved targeting of the CNS [168]. Emerging evidence highlights Nutritional Psychiatry and flavonoid-rich dietary patterns as accessible, sustainable interventions for global mental health promotion [162]. Flavonoids represent potent, multifunctional neuroimmune modulators and act as a crucial nexus connecting nutrition, neuroscience, and precision medicine. Their validation within rigorous translational frameworks is essential to establish Nutritional Psychiatry as a sustainable and accessible therapeutic strategy for global mental health.

## 7. Conclusions

Affective disorders are among the most significant challenges in global mental health, with their pathophysiology now recognized as multidimensional, involving persistent neuroinflammation, oxidative stress, impaired neurotrophic signaling, and disruption of the MGB axis. The evidence reviewed here highlights the potential of flavonoids and structurally related natural compounds as multi-target modulators of these converging pathological mechanisms.

Mechanistically, compounds such as quercetin, luteolin, apigenin, chrysin, and EGCG consistently suppress neuroinflammatory cascades by inhibiting the NF-κB/NLRP3 inflammasome axis and reducing pro-inflammatory cytokines, including TNF-α, IL-1β, and IL-6. At the same time, these compounds reduce oxidative damage by activating the Nrf2-ARE antioxidant pathway, promote neuroplasticity through upregulation of BDNF/TrkB signaling, and modulate monoaminergic and GABAergic neurotransmission, thereby addressing multiple pathophysiological features of anxiety and depression simultaneously.

Preclinical evidence is robust and consistent, with numerous validated animal models of chronic stress, LPS-induced neuroinflammation, and pharmacological paradigms demonstrating significant anxiolytic and antidepressant effects following flavonoid administration. Notably, quercetin and luteolin have shown the most extensive preclinical characterization, with documented dose-dependent improvements in behavioral phenotypes and corresponding reductions in neuroinflammatory biomarkers. In the context of BD, preclinical data further support the relevance of flavonoids in addressing the interplay between neuroinflammation, oxidative stress, and mitochondrial dysfunction that drives neuroprogression in this population.

From a nutritional perspective, certain foods are notable for containing the greatest diversity of flavonoid subclasses, making them especially relevant as dietary recommendations for supporting neurological and emotional health. Berries—including blueberries, blackberries, strawberries, and raspberries—are among the richest and most varied sources, providing anthocyanins (cyanidin, malvidin, delphinidin), flavonols (quercetin, kaempferol), flavanols (catechin, epicatechin), and phenolic acids with documented neuroprotective activity. Red and purple grapes, as well as grape-derived products such as red wine, also offer a broad range of flavonoid subclasses, including anthocyanins, flavonols, flavanols, and stilbenes such as resveratrol. Green tea (*Camellia sinensis*) is another exceptional source, particularly rich in flavan-3-ols—notably EGCG—along with flavonols and phenolic acids. Citrus fruits (oranges, grapefruits, lemons) provide flavanones (hesperidin, naringenin, eriocitrin) together with flavones and flavonols, and contribute to the Mediterranean dietary pattern associated with reduced depression risk. Legumes, especially soybeans and their derivatives, are the primary source of isoflavones (genistein, daidzein), which complement the flavonoid spectrum of mixed diets. Herbs such as parsley, celery, and chamomile are notable sources of flavones (apigenin, luteolin) with well-characterized anxiolytic and anti-inflammatory properties. In practical terms, dietary patterns that combine these food groups—as exemplified by the Mediterranean diet and flavonoid-rich plant-based approaches—are most likely to ensure adequate intake across multiple flavonoid subclasses, thereby engaging the widest range of neuroprotective mechanisms simultaneously.

In contrast, clinical evidence remains limited, heterogeneous, and frequently inconclusive. Although some trials have demonstrated beneficial effects of flavonoid-rich dietary interventions and isolated compounds on depressive and anxiety symptoms, particularly in populations with comorbid inflammatory or metabolic conditions, the anti-inflammatory mechanisms underlying these improvements are not consistently verified. Methodological variability across studies, including differences in dosing regimens, formulation strategies, participant characteristics, and outcome measures, substantially constrains the generalizability and interpretability of available findings.

Several critical challenges must be addressed to advance the clinical translation of flavonoid-based therapeutics. First, the poor oral bioavailability of most flavonoids, resulting from low aqueous solubility, extensive first-pass metabolism, and limited BBB penetration, constitutes a fundamental pharmacokinetic barrier. Nanotechnology-based delivery platforms, including polymeric nanoparticles, liposomes, and nanogels, represent promising strategies to overcome these limitations, as evidenced by superior efficacy of nanoformulated quercetin and curcumin in preclinical models. Second, the potential for pharmacokinetic interactions between flavonoids and cytochrome P450 enzymes necessitates careful evaluation in clinical populations receiving polypharmacy, particularly patients with BD on mood-stabilizing regimens. Third, the heterogeneity of affective disorder populations including differences in inflammatory status, illness phase, and comorbidities highlights the need for biomarker-guided stratification in future clinical trials to identify those patient subgroups most likely to benefit from flavonoid-based interventions.

Beyond pharmacological approaches, Nutritional Psychiatry offers a complementary framework in which flavonoid-rich dietary patterns, including the Mediterranean diet and targeted supplementation with berry-derived anthocyanins, may confer preventive and adjunctive benefits for affective disorders at a population level. The modulation of the MGB axis by flavonoids and their colonic metabolites further expands the potential therapeutic reach of these compounds, as emerging evidence links gut microbiota composition to neuroinflammatory tone and mood regulation.

In conclusion, flavonoids represent a scientifically compelling and pharmacologically versatile class of neuroimmune modulators with documented preclinical efficacy and mechanistic plausibility for the management of neuroinflammation-driven affective disorders. However, the translation of these findings into clinical practice demands rigorous, large-scale randomized controlled trials with bioavailability-optimized formulations, standardized dosing protocols, validated neuropsychiatric and inflammatory endpoints, and careful assessment of drug-flavonoid interactions. The integration of systems pharmacology, omics-based approaches, and precision medicine principles will be essential to fully realize the therapeutic potential of these compounds and to establish Nutritional Psychiatry as a sustainable, evidence-based strategy for addressing the global burden of affective disorders.

## Figures and Tables

**Table 1:** Effects of flavonoids on neuroinflammation, anxiety and depression in animal models.

Flavonoid	Animal Model	Dosage and Time of Treatment	Behavioral Data	Biomarkers Evaluated	Possible Mechanism Involved	References
Bioactive Dietary Polyphenol Preparation	Mice (chronic stress induced)	62 mg/kg for 2 weeks.	Significantly decreased depression-like and anxiety-like behaviors; locomotor activity unaffected.	Microglia activation (reversed from amoeboid to ramified shape) in amygdala and hippocampal formation.	Modulating regional heterogeneity of microglia morphology.	[116]
Narirutin	Mice	1 week treatment	Significantly alleviated depressive-like behaviors, indicated by restored decreased sucrose preference and shortened floating time in FST.	Not explicitly detailed in excerpt.	Antioxidant and anti-inflammatory activities (general properties of Narirutin).	[117]
Chrysin	Pre-clinical research (implied rodents)	5 and 20 mg/kg for 28 days.	Anxiolytic- and antidepressant-like effects.	Neurotransmitter systems, neurotrophic factors.	Interaction with GABAergic and serotonergic neurotransmitter systems; activation of neurotrophic factors; antioxidant and anti-inflammatory activities.	[106]
Helichrysum stoechas Methanolic Extract (contains flavonoids)	Mice (anxiety-related environment)	30, 60 and 100 mg/kg for 2 weeks.	Dose-dependent anxiolytic-like activity in light dark box and marble burying tests; no antidepressant-like activity in tail suspension test; no sedative/motor impairment.	Phosphorylation of ERK44/42 (counteracted reduction); BDNF expression (restored); CREB levels (returned to basal) in noradrenergic hippocampal neurons.	Upregulation of ERK signaling pathways; restoration of BDNF and CREB levels.	[118]
Saffron extract (Safr’Inside™) (contains carotenoids)	Mouse model of low-grade inflammation	4.5 mg/kg for 2 months.	Improved anxiety-related behavior.	Gut microbiota speciation (16S rRNA sequencing); gut metabolites (1H-NMR); brain proteomic analysis.	Modulation of microbiota and gut-derived metabolites; modulation of monoaminergic neurotransmission and oxidative stress.	[119]
Quercetin	Male mice (chronic restraint stress and LPS-induced anxiety models)	50 mg/kg for 2 weeks.	Ameliorates CRS- and LPS-induced anxiety-like behaviors.	Neuroinflammation in the lateral hypothalamus and bed nucleus of the stria terminalis.	Modulating neuroinflammation in luteinizing hormone (LH) and core of the bed of the stria terminalis (BNST).	[120]
Pinocembrin	CUMS mouse model	10 mg/kg for 3 weeks.	Alleviated decreased sucrose preference/body weight; reversed increased immobility time in FST/TST; reduced crossing/rearing score in OFT.	ROS, malondialdehyde (MDA), superoxide dismutase; inflammatory factors (IL-1β, TNF-α, IL-10, TGF-β).	Ameliorating neuroinflammation and apoptosis.	[114]
Quercetin	Corticosterone-induced mice (depression-like behaviors)	40 and 80 mg/kg for 2 weeks	Mitigates depression-like behavior.	IL-1β, IL-6, TNF-α, MM-0132M2, GSH, GST, CAT, NO, ALT, ALP and AST.	Suppression of neuroinflammation and oxidative damage.	[121]
Quercetin (in BDNF-alginate nanogels)	Reserpine-induced rats, stress-induced mice, CUMS rats	20 mg/kg for 2 days.	Antidepressant effects on reserpine-induced rats; reversed despair behavior in stress-induced mice; antidepressant effects on CUMS rats.	BDNF, IL-6 and PGE2	Enhanced bioavailability, rapid brain distribution, delivery of BDNF.	[122]
Quercetin	LPS-induced rats (depression-like behavior)	40 kg/kg for 2 weeks	Alleviates LPS-induced depression-like behavior.	BDNF-related imbalance of Copine 6 and TREM1/2 in the Hippocampus and prefrontal cortex (PFC).	Regulating BDNF-related imbalance of Copine 6 and TREM1/2 in the Hippocampus and PFC.	[123]
Scopoletin (a coumarin)	CFA-induced mouse model (chronic inflammation anxiety)	2.0, 10.0, 50.0 mg/kg for 2 weeks.	Dose-dependently ameliorated CFA-induced anxiety-like behaviors in open field test and elevated plus maze test (EPM).	Microglia activation; peripheral/central IL-1β, IL-6, TNF-α levels; excitatory/inhibitory receptors and neurotransmitters.	Inhibition of NF-κB and MAPK signaling pathways; anti-inflammatory activities; regulation of excitatory/inhibitory balance.	[124]
Luteolin	Rats subjected to sleep deprivation	10 and 20 mg/kg for 3 weeks.	Reversed anxiety and depressive-like behavior.	NF-κB, ASC, NLRP3, and active Casp-1 in the HC	Modulation of the NF-κB/NLRP3 inflammasome axis in the hippocampus.	[32]

## Data Availability

No new data were created or analyzed in this study.

## Abbreviations

The following abbreviations are used in this manuscript:

BBB	Blood–brain barrier
4 MyD88	4-myeloid differentiation primary response 88
5-HT	Serotonin
8-OHdG	8-hydroxy-2′-deoxyguanosine
AKT	Protein kinase B
ALP	Alkaline phosphatase
ALT	Alanine aminotransferase
AMPK	AMP-activated protein kinase
ARE	Antioxidant response element
ASC	Apoptosis-associated speck-like protein containing a CARD
AST	Aspartate aminotransferase
BDNF	Brain-derived neurotrophic factor
BNST	Bed nucleus of the stria terminalis
BUN	Blood urea nitrogen
BV2	BV2 microglial cells
CARD	Caspase recruitment domain
CAT	Catalase
CB_1_	Cannabinoid receptor type 1
CB_2_	Cannabinoid receptor type 2
CNS	Central nervous system
COMT	Catechols-O-methyltransferase
COX-2	Cyclooxygenase-2
CREB	cAMP response element-binding protein
CRP	C-reactive protein
CRS	Chronic restraint stress
CUMS	Chronic unpredictable mild stress
D1	Dopamine receptor D1
D2	Dopamine receptor D2
DA	Dopamine
DCX	Doublecortin
DSS	Dextran sulfate sodium
EGCG	Epigallocatechin-3-gallate
eIF2α	Eukaryotic initiation factor 2 alpha
EPM	Elevated plus maze test
ERK	Extracellular signal-regulated kinase
FOS	Proto-oncogene c-Fos
FST	Forced swim test
GABA	Gamma-aminobutyric acid
GBB	Gut-blood barrier
GDNF	Glial cell-derived neurotrophic factor
GOS	Galacto-oligosaccharides
GPx	Glutathione peroxidase
GRP78	Glucose-regulated protein 78
GSH	Glutathione
GST	Glutathione S-transferase
HC	Hippocampus
HMGB1	High Mobility Group Box 1
HO-1	Heme oxygenase-1
HPA	Hypothalamic–pituitary–adrenal axis
HSP90	Heat shock protein 90
ICAM-1	Intercellular adhesion molecule 1
IFNγ	Interferon gamma
IL-10	Interleukin-10
IL-17	Interleukin-17
IL-1β	Interleukin-1β
IL-6	Interleukin-6
iNOS	Inducible nitric oxide synthase
IRAK	Interleukin-1 receptor-associated kinase
JAK	Janus kinase
JNK	c-Jun N-terminal kinase
LDL	Low-density lipoprotein
LH	Luteinizing hormone
LPS	Lipopolysaccharides
M1	Pro-inflammatory
M2	Anti-inflamatory
MAO	Monoamine oxidase
MAO-A	Monoamine oxidase A
MAOIs	Monoamine oxidase inhibitors
MAPK	Mitogen-activated protein kinase
MDA	Malondialdehyde
MDSC	Myeloid-derived suppressor cells
MGB	Microbiota-gut–brain axis
MKK4	Mitogen-activated protein kinase kinase 4
MPO	Myeloperoxidase
NE	Norepinephrine
NF-κB	Nuclear factor kappa B
NF-κB/p65	Nuclear factor kappa B/p65 subunit
NGF	Neuronal growth factor
NK	Natural killer
NLRP3	NLR family pyrin domain containing 3
NO	Nitric oxide
Nrf2	Nuclear factor erythroid 2–related factor 2
OFT	Open field test
PFC	Prefrontal cortex
PGC1α	Peroxisome proliferator-activated receptor gamma coactivator 1-alpha
PGE2	Prostaglandin E2
PI3K	Phosphoinositide 3-kinase
PPAR-γ	Peroxisome proliferator-activated receptor gamma
RAGE	Receptor for Advanced Glycation End-products
ROS	Reactive oxygen species
SCFAs	Short-chain fatty acids
SIRT1	Sirtuin 1
SLC6A4	Solute carrier family 6 member 4
SOD	Superoxide dismutase
SPS	Single prolonged stress
SSRIs	Selective serotonin reuptake inhibitors
STAT	Signal transducer and activator of transcription
TBARS	Thiobarbituric acid-reactive substances
TGF-β	Transforming growth factor beta
TLR2	Toll-like receptor 2
TLR4	Toll-like receptor 4
TNF-α	Tumor necrosis factor alpha
Treg	Regulatory T-cells
TrkB	Tropomyosin receptor kinase B
TST	Tail suspension test
Us-CRP	Ultra-sensitive C-reactive protein
VCAM-1	Vascular adhesion molecule 1
VLDL	Very-low-density lipoprotein
Wnt/β-Catenin	Wnt/beta-catenin signaling pathway
β-catenin	Beta-catenin
